# Tetralogy of Fallot: Stroke in a Young Patient

**DOI:** 10.7759/cureus.2714

**Published:** 2018-05-31

**Authors:** Hassam Ali, Shiza Sarfraz, Muhammad Sanan

**Affiliations:** 1 Medical 4, Bahawal Victoria Hospital, Quaid-E-Azam Medical College, Bahawalpur, PAK; 2 Department of Anesthesiology, Bahawal Victoria Hospital, Quaid-E-Azam Medical College, Bahawalpur, PAK

**Keywords:** congenital heart defects, embolic stroke, tetralogy of fallot, paralysis, cardiology, cerebrovascular accident, heart, neurology, international medicine

## Abstract

Tetralogy of Fallot (TOF) is a congenital birth defect of the heart which actually comprises four individual flaws. It causes poor flow of oxygenated blood to the organs and leads to cyanosis (blue-tinted skin, because of inadequate oxygenation). It can be recognized at birth or in adulthood. But sometimes, cases may go unnoticed, and the patient might present with some rare complications. In this case, the patient presented with an embolic infarct of the brain at the age of 25 with an undiagnosed tetralogy of Fallot.

## Introduction

Tetralogy of Fallot (TOF) is a heart disease present at birth [1]. It consists of four defects [2]:

- Pulmonary stenosis

- Ventricular septal defect

- Right ventricular hypertrophy

- Aorta overriding the ventricular septal defect

Some babies, when they cry or breastfeed, may turn very blue. This is because of "a tet spell" due to the shunting of excess deoxygenated blood from the right chamber of the heart to the left chamber. Such babies may have difficulty breathing, become limp, or even lose consciousness [3].

TOF is treated by open-heart surgery, mostly in the first year of life. Much of the treatment plan depends upon the individual’s signs and symptoms [4]. Most individuals can live to become adults, though complications may arise later—including irregular heart rates, cerebrovascular accidents [5], and pulmonary regurgitation. We hereby present a case of a female with an undiagnosed tetralogy of Fallot, presenting with an embolic stroke of the frontoparietal region of the brain. Informed consent was obtained from the patient.

## Case presentation

A 25-year-old female presented to the emergency room with a complaint of left-sided body weakness since 12 hours. On clinical examination, the power of the left upper and lower limbs was seen to be limited to just slight movement. Planter reflex was up going on the left side (Babinski positive). Clinical anemia was also present, and the nails showed massive clubbing. According to her parents, she had a history of cyanosis since birth, but they never got treatment for it. There was no history of any psychiatric illness, hypertension, or diabetes. 

**Figure 1:**
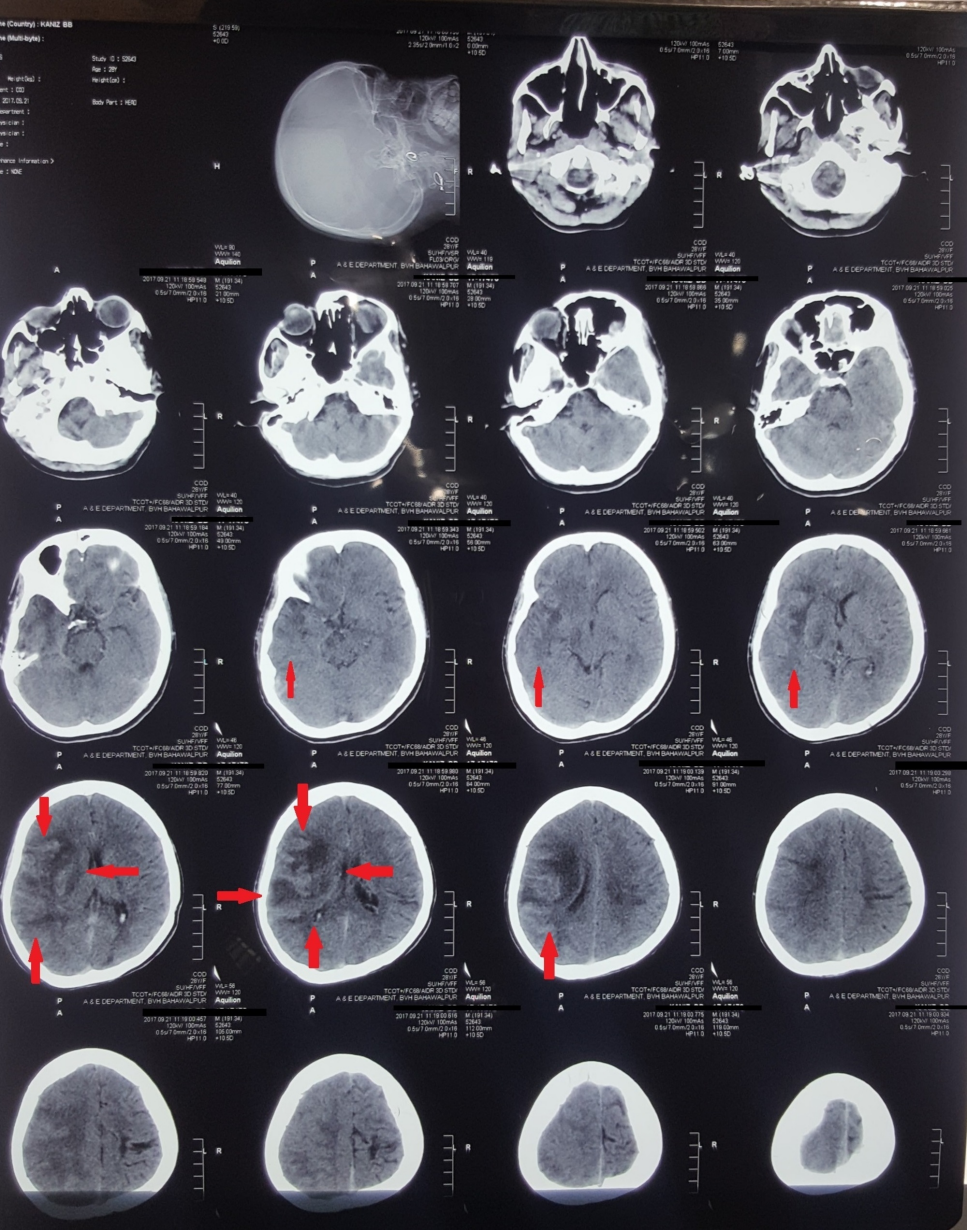
Computed tomography (CT) scan showing changes in the right temporoparietal region

**Figure 2:**
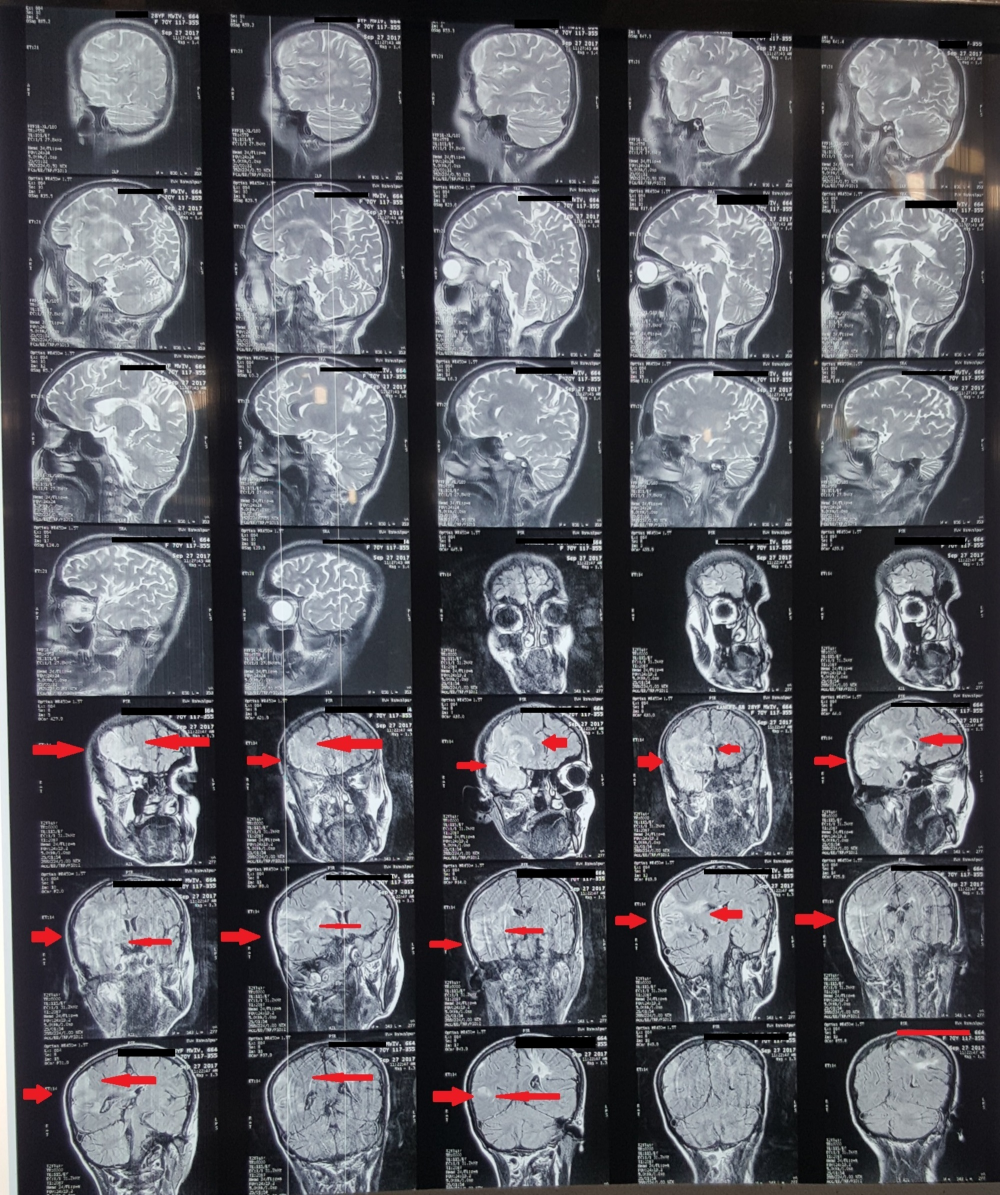
Magnetic resonance imaging (MRI) showing changes in the infarct (red arrows)

**Figure 3:**
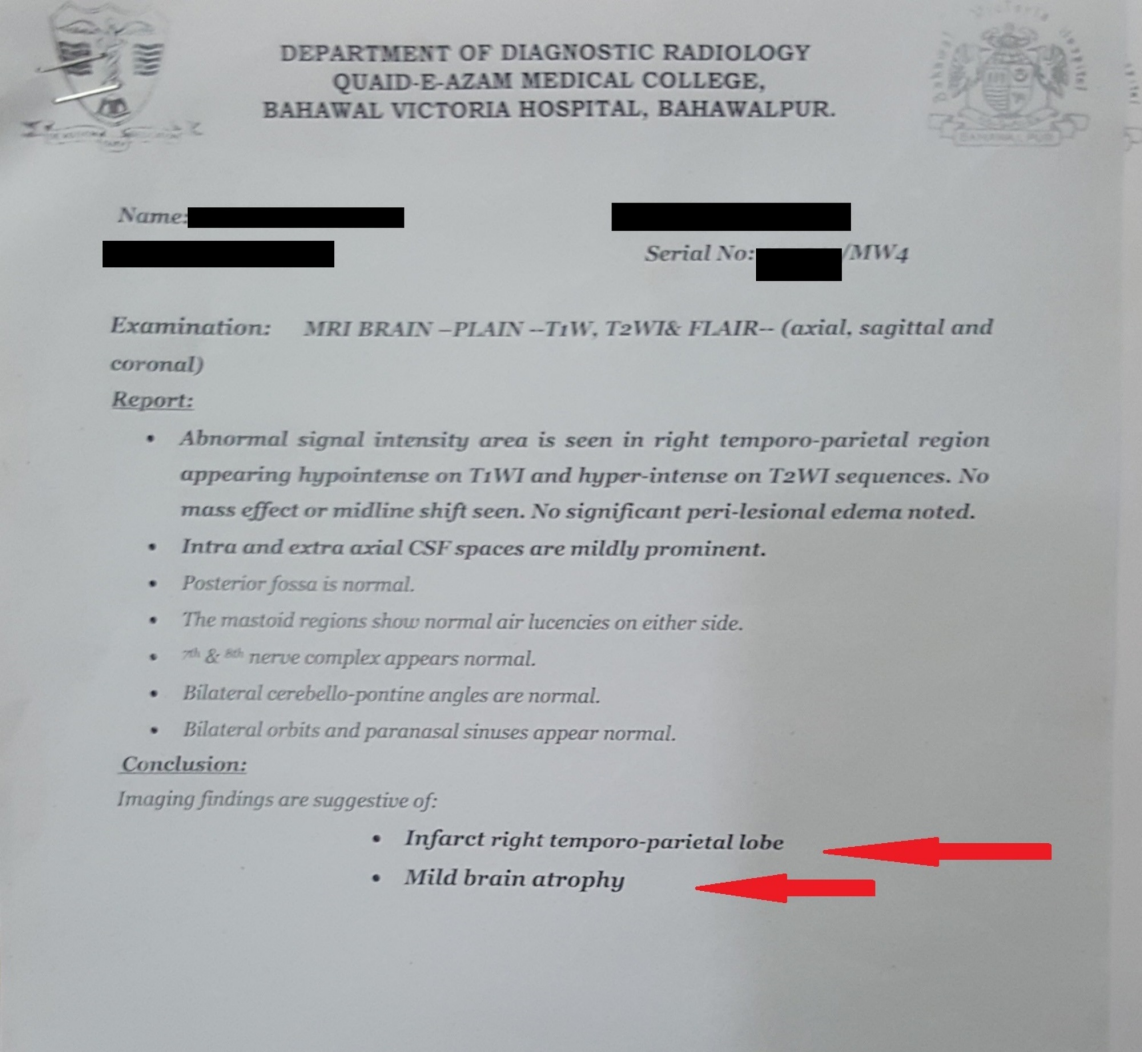
Magnetic resonance imaging report

A CT (computed tomography) scan showed no evidence of a haemorrhage, but some changes in the temporoparietal area were observed, as shown in Figure 1. Later, an MRI (magnetic resonance imaging) with contrast was advised and performed, which showed an infarct of the right temporoparietal lobe with mild brain atrophy as shown in Figure 2 and Figure 3.

## Discussion

**Figure 4:**
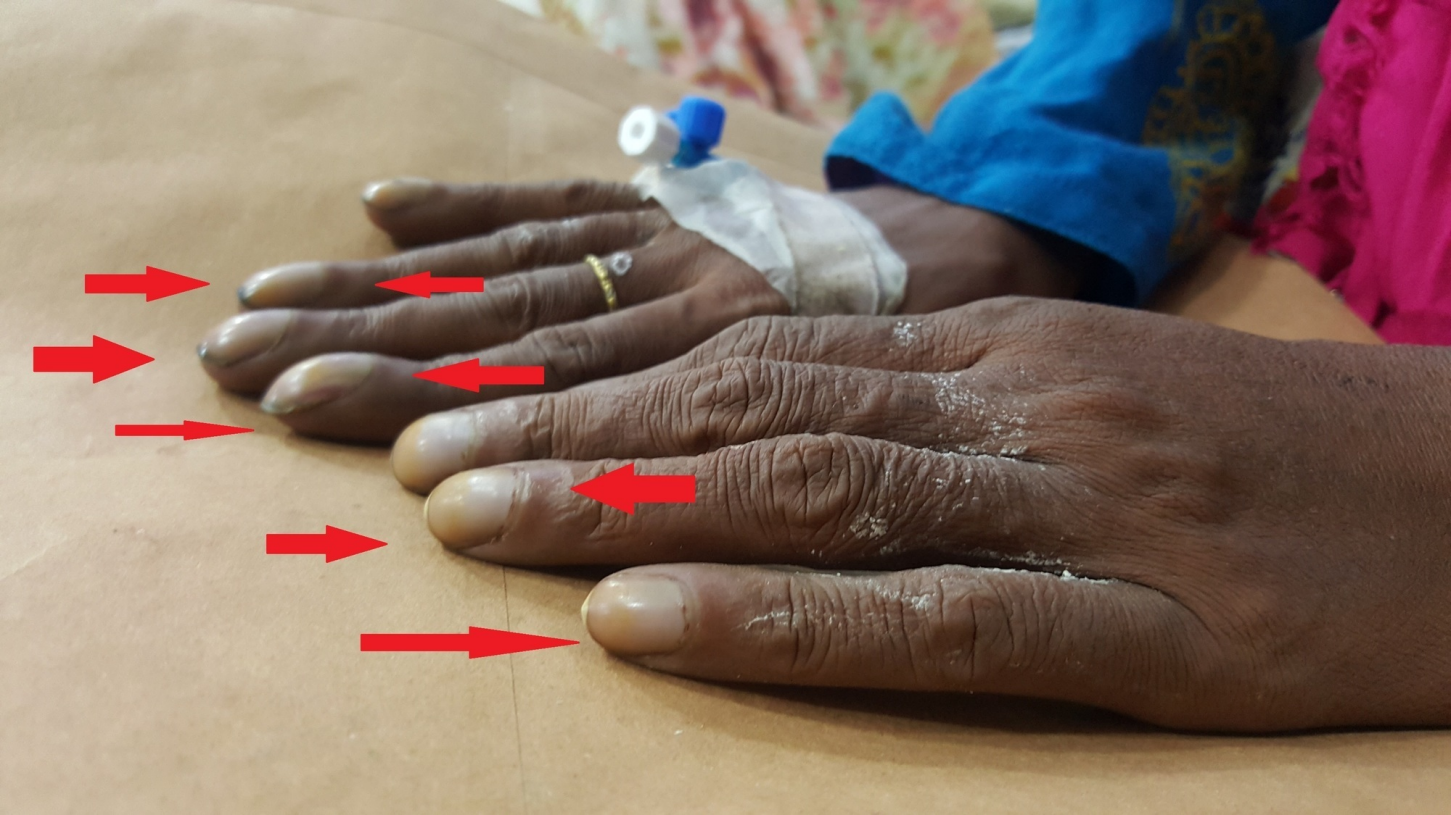
Finger nails clubbing of patient

**Figure 5:**
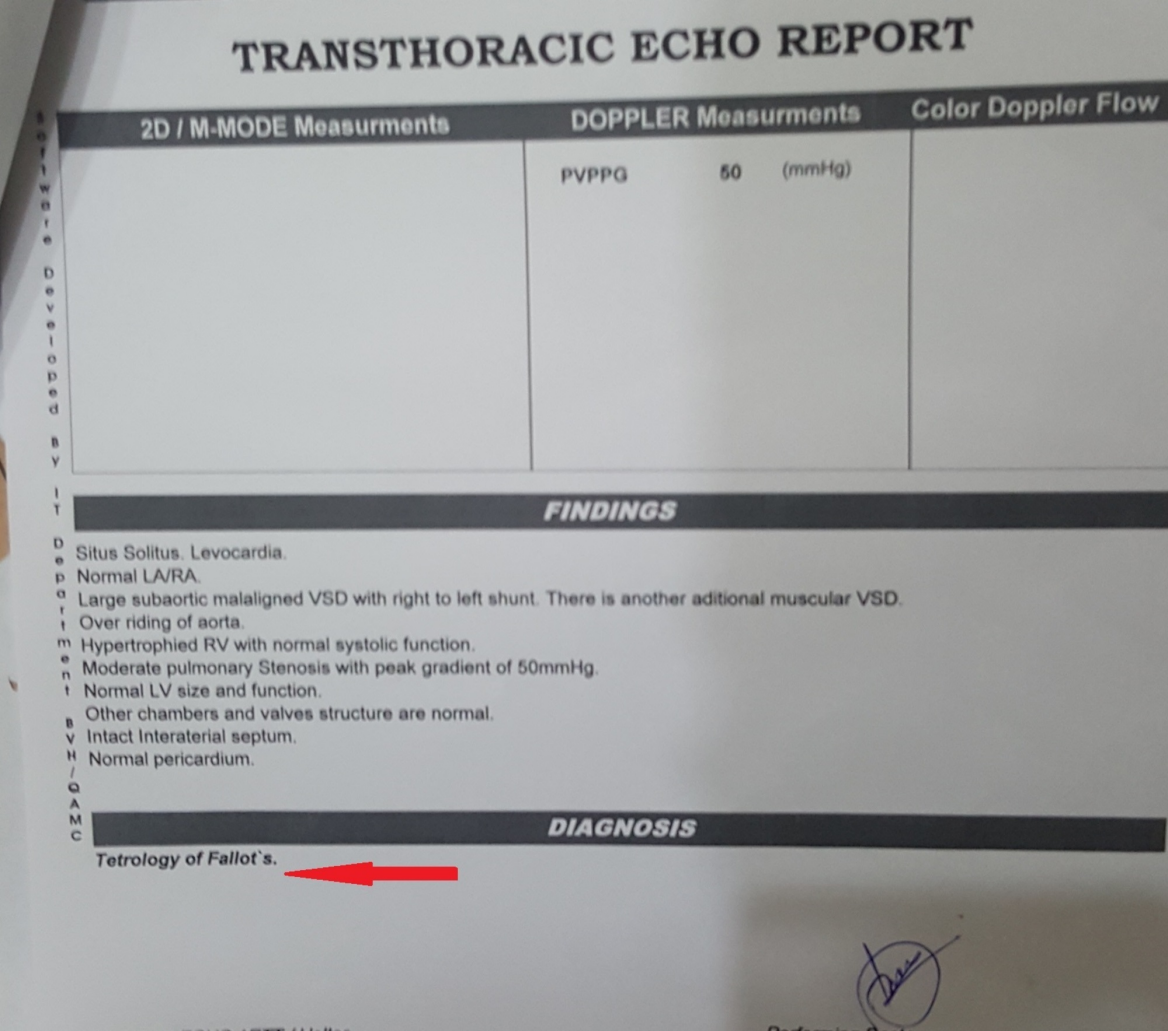
Echocardiography report

Cerebrovascular accidents (CVAs) are not very common in young patients, but the patient was from a village where medical facilities were not present. She was never diagnosed with tetralogy of Fallot, and only presented to the emergency room with left-sided paralysis as in a stroke. She did have physical manifestations of chronic disease like emaciation [6] and clubbing of fingers as shown in Figure 4, which made us consider some underlying heart etiology. Later, her echocardiography report (Figure 5) confirmed a structural heart disease.

Congenital cardiac disease can lead to the formation of a thrombus inside the heart, which can later throw clots as emboli into the peripheral circulation; one study even suggested that congenital heart disease has a role in increasing thrombogenicity [7], which we also suspected in this patient.

Alioglu et al. reported the occurrence of intra-cardiac thrombosis (two in right atrium and one in right ventricle) in three of nine children with tetralogy of Fallot [8]. Ammash et al. reported two cases of cerebrovascular embolism among eight patients diagnosed with TOF over a seven-year period [9]. Ammash's study was the first to show the association between cyanotic congenital heart disease and stroke in adults.

There are several factors that increase risk of pathogenesis of thrombosis in patients with congenital heart disease (CHD). For example, chronic acidosis increases fibrin deposition, secondary erythrocytosis, and hypoxia/hypoxemia-induced activation of the pro-coagulant pathways, increasing tissue factor expression and impaired fibrinolysis [8]. Adults with cyanotic CHD also have an increased red blood cell (RBC) mass. This secondary erythrocytosis may increase blood viscosity, and it may thereby reduce cerebral blood flow, which can predispose the patient to clot formations. Chronic hypoxemia will also activate neutrophils and mononuclear cells that release vasoactive and chemotactic factors, resulting in endothelial injury [8]. Platelets and endothelial cells interact and activate platelets and enhance intravascular thrombus formation by thrombin, which activates the coagulation cascade. In addition, an impaired fibrinolytic system due to increased plasminogen activator-1 levels can contribute to thrombogenicity [8] and not just blood turbulence; thus, all these factors should also be taken under consideration for cerebrovascular accidents in such patients. This may lead to future use of anticoagulation drugs in such patients in addition to surgical correction. 

Due to advances in medical fields and paedriatic surgery, many cases of tetralogy of Fallot (TOF) undergo prompt surgical treatment. But our patient was born in a remote village with little or no medical facilities, and her diagnosis of TOF was delayed due to illiteracy as well as quackery in that region, though much research is needed to link causation of these factors. For our ward, this was the first case of stroke in such a young patient due to TOF.

## Conclusions

Cerebrovascular accidents are usually seen in patients with a history of hypertension, diabetes, or structural cardiac anomalies—more so in older patients than in the young. Although it is not common, stroke in young patients can have multiple etiologies, including an undiagnosed congenital heart disease that can lead to the formation of clots and their discharge into the peripheral circulation, leading to a blockade of that area's blood supply. Our case report followed a somewhat similar course where tetralogy of Fallot was the culprit that caused a stroke in a young patient. The patient presented with left-sided body paralysis, which upon investigation lead to a diagnosis of tetralogy of Fallot. We emphasize that structural heart diseases should be kept in the differential for strokes, especially in younger people. It will help in early diagnosis and treatment.
